# Serotonin Transporter Deficiency is Associated with Dysbiosis and Changes in Metabolic Function of the Mouse Intestinal Microbiome

**DOI:** 10.1038/s41598-019-38489-8

**Published:** 2019-02-14

**Authors:** Megha Singhal, Benjamin A. Turturice, Christopher R. Manzella, Ravi Ranjan, Ahmed A. Metwally, Juliana Theorell, Yue Huang, Waddah A. Alrefai, Pradeep K. Dudeja, Patricia W. Finn, David L. Perkins, Ravinder K. Gill

**Affiliations:** 1 Division of Gastroenterology & Hepatology, University of Illinois at Chicago, Chicago, USA; 2 Division of Pulmonary, Critical Care, Sleep and Allergy, University of Illinois at Chicago, Chicago, USA; 3 Department of Microbiology & Immunology, University of Illinois at Chicago, Chicago, USA; 4 Department of Physiology and Biophysics, University of Illinois at Chicago, Chicago, USA; 5 Division of Nephrology, University of Illinois at Chicago, Chicago, USA; 6 Department of Surgery, University of Illinois at Chicago, Chicago, USA; 7 Department of Bioengineering, University of Illinois at Chicago, Chicago, USA; 8 Jesse Brown VA Medical Center, Chicago, IL USA

## Abstract

Serotonin transporter (SERT) plays a critical role in regulating extracellular availability of serotonin (5-HT) in the gut and brain. Mice with deletion of SERT develop metabolic syndrome as they age. Changes in the gut microbiota are being increasingly implicated in Metabolic Syndrome and Diabetes. To investigate the relationship between the gut microbiome and SERT, this study assessed the fecal and cecal microbiome profile of 11 to 12 week-old SERT^+/+^ and SERT^−/−^ mice. Microbial DNA was isolated, processed for metagenomics shotgun sequencing, and taxonomic and functional profiles were assessed. 34 differentially abundant bacterial species were identified between SERT^+/+^ and SERT^−/−^. SERT^−/−^ mice displayed higher abundances of Bacilli species including genera *Lactobacillus, Streptococcus, Enterococcus*, and *Listeria*. Furthermore, SERT^−/−^ mice exhibited significantly lower abundances of *Bifidobacterium* species and *Akkermansia muciniphilia*. Bacterial community structure was altered in SERT^−/−^ mice. Differential abundance of bacteria was correlated with changes in host gene expression. *Bifidobacterium* and *Bacilli* species exhibited significant associations with host genes involved in lipid metabolism pathways. Our results show that SERT deletion is associated with dysbiosis similar to that observed in obesity. This study contributes to the understanding as to how changes in gut microbiota are associated with metabolic phenotype seen in SERT deficiency.

## Introduction

Serotonin (5-HT) is an important neurotransmitter in the central nervous system. However, approximately 95% of total body 5-HT is present in the gastrointestinal (GI) tract. 5-HT has diverse roles in the GI tract which include regulating mucosal homeostasis, increasing motility, and modulating electrolyte transport^1–7^. Optimal levels of 5-HT are maintained via the serotonin transporter (SLC6A4, SERT) which actively imports 5-HT, allowing for its degradation by intracellular monoamine oxidases^8,9^. Additionally, circulating platelets uptake 5-HT from the GI tract via SERT which allows distribution of gut-derived 5-HT to peripheral tissues where it regulates bone remodeling, coagulation, and energy metabolism^10–12^. Both blood plasma and platelets are depleted of 5-HT in SERT^−/−^ mice^1,13^. In addition, most peripheral organs have significantly depleted 5-HT levels, confirming that their normal serotonin content is dependent upon SERT^1,14–18^. SERT^−/−^ mice, thus, exhibit a pleotropic phenotype which includes anxiety-like behavior, reduced bone density and strength, and GI abnormalities^19,20^.

In addition to other observed abnormalities, there is a metabolic phenotype similar to Type-2 diabetes (T2D) that develops in SERT^−/−^ mice as they reach adulthood^19,21,22^. At 3 months of age, there is no significant difference in body weight in SERT^−/−^ vs. SERT^+/+^ mice, but an increase in visceral adiposity with a decrease in brown adipose tissue is observed in SERT^−/−^ mice^21^. Additionally, SERT^−/−^ mice exhibit many metabolic disturbances at this age including hyperinsulinemia, hyperleptinemia, and insulin resistance, prior to any measurable weight gain^22^. These effects are independent of food consumption, but a decrease in locomotor activity has not been definitively ruled out as a possible cause^19,21,22^. Selective serotonin reuptake inhibitors (SSRIs) are pharmacological agents which inhibit SERT function that are used to treat depression and other psychiatric disorders. In accordance with the metabolic phenotype in SERT^−/−^ mice, patients taking SSRIs exhibit weight gain and an increased risk of developing T2D^23^. Therefore, it is critical to enhance our understanding of how decreased SERT function is linked to metabolic disorders.

Increasing evidence implicates the role of the gut microbiome, which refers to the functional network of microbes that reside inside the gut, in regulating normal host physiology as well as pathophysiology. For example, gut microbes secrete various molecules, breakdown non-digestible macromolecules, and stimulate the immune system^24^. As the gut microbiota reside at the interface of ingested nutrients and the host, it is not surprising that many studies have linked changes in gut microbiome to metabolic syndrome. For example, administration of broad spectrum antibiotics to obese mice ameliorate glucose intolerance independent of food intake or adiposity^25^. In both mouse-models of metabolic syndrome and patients with T2D, the abundances of *Akkermansia muciniphila* and *Bifidobacteria* are decreased^26^. These bacteria are thought to protect against leakage of bacterial products from gut bacteria into the bloodstream as a result of increased intestinal permeability, a proposed mechanism by which gut microbiota contribute to the development of metabolic syndrome^26^.

While it is known that gut bacteria are able to influence host serotonergic machinery^27–30^, the effect of changes in host serotonergic signaling on intestinal microbiome homeostasis has not been investigated in mice. We hypothesized that SERT deletion in mice is associated with changes in the composition and function of bacteria in the intestine. Utilizing the fecal and cecal stool samples from global SERT^−/−^ mice and SERT^+/+^ littermates, we analyzed the impact of SERT deficiency on the intestinal microbiome. Our results show that deletion of SERT in mice causes dysbiosis. Further, we found that bacterial community structure is disrupted, and metabolic capabilities of the microbiome are altered in SERT^−/−^ mice. Based upon our findings from our previous microarray analysis comparing gene expression in the ileal mucosa of SERT^+/+^ and SERT^−/−^ mice (GEO accession GSE93534), we correlated host expression of enzymes involved in lipid metabolism with changes in gut microbiome^31^. Indeed, expression of these enzymes correlated with changes in gut bacterial metabolism of unsaturated fatty acids. These studies enlighten our understanding of how dysregulation of the host serotonergic system can have a significant impact on the intestinal microbiome, which in-turn likely impacts overall host physiology.

## Results

### β-diversity, but not α-diversity, is altered by SERT deficiency

**Figure 1:**
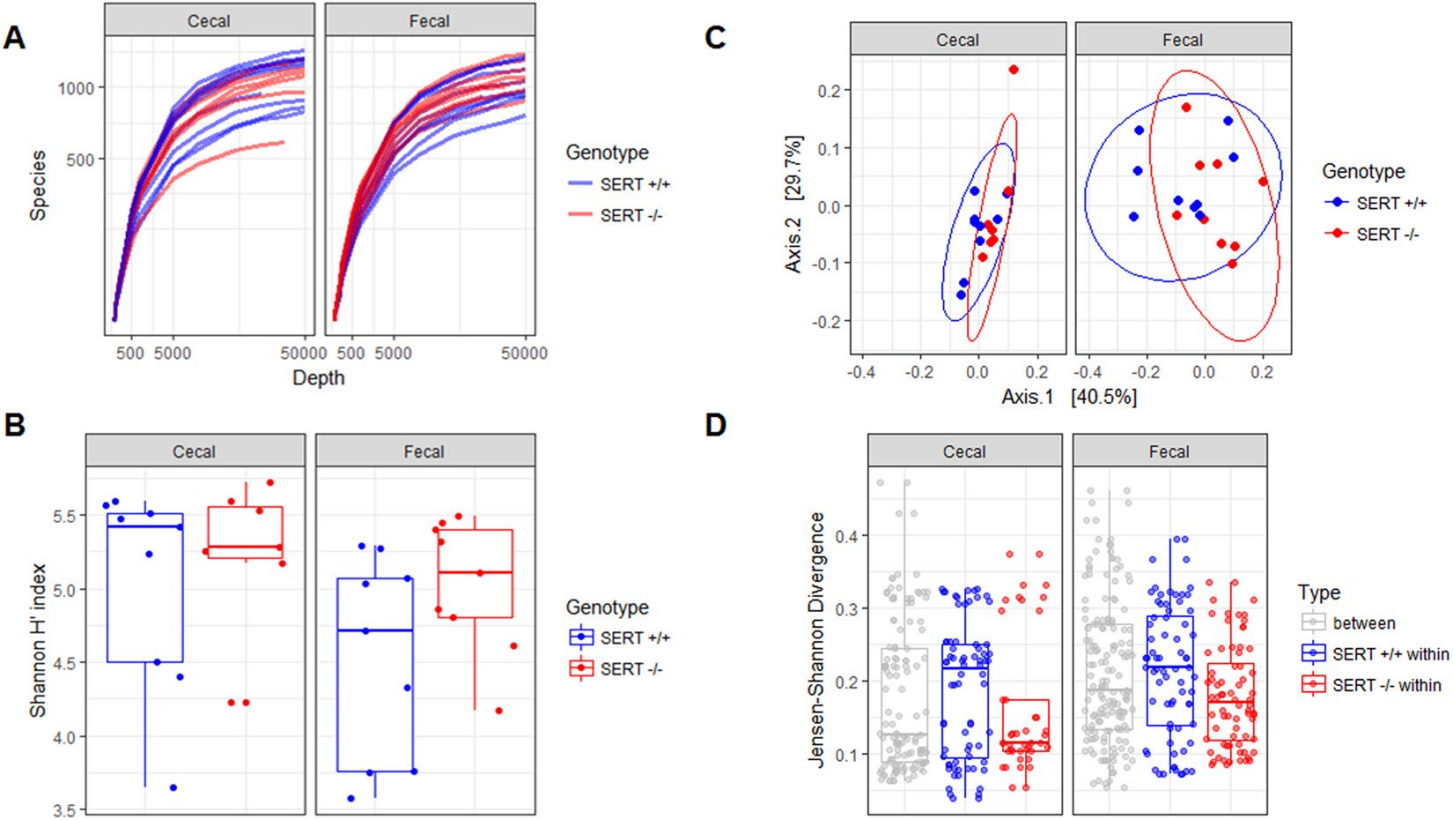
SERT deficiency alters bacterial diversity. (**A**) Rarefaction curves between SERT^+/+^ and SERT^−/−^ cecal and fecal samples. (**B**) Box-plot of α-diversity (Shannon index) between SERT^+/+^ and SERT^−/−^ cecal and fecal samples. Differential testing by two-way ANOVA, *p* < 0.05 was considered significant. (**C**) PCoA, displaying the first two principle components, of β-diversity (Jensen-Shannon divergence) between SERT^+/+^ and SERT^−/−^ cecal and fecal samples. Differential testing PERMANOVA using *adonis*, *p* < 0.05 was considered significant. (**D**) Jensen-Shannon Divergence between and within strains in fecal and cecal samples.

To assess differences in the ecological structure of the bacterial populations in each mouse’s cecal and fecal isolates between SERT^−/−^ and SERT^+/+^ mice, α- and β- diversity were determined. There was no significant difference (*p* > 0.05, Two-way ANOVA) in the α-diversity of SERT^−/−^ mice as compared to SERT^+/+^ littermates as calculated by rarefaction curve and Shannon diversity index (Fig. 1A,B). β-diversity was significantly different (*p* < 0.05, PERMANOVA) between SERT^−/−^ and SERT^+/+^ mice and trended (*p* = 0.05, PERMANOVA) towards a difference between fecal and cecal samples as measured by Jensen-Shannon divergence (Fig. 1C). There were no significant differences between the two batches of mice. Notably, the difference between SERT^−/−^ and SERT^+/+^ mice accounts for 9% of variance in microbial abundances (r^2^ = 0.09, PERMANOVA), indicating the differences between the SERT^−/−^ and SERT^+/+^ mice are significant but not large. Jensen-Shannon Divergence was compared between and within strains in fecal and cecal samples (Fig. 1D). SERT^−/−^ had lower with-in group variance as compared to SERT^+/+^ mice, indicating that SERT deficiency has a selective pressure on the microbiota.

### SERT deficiency changes the abundance of Firmicutes, Actinobacteria, and Verrucomicrobia species

**Figure 2:**
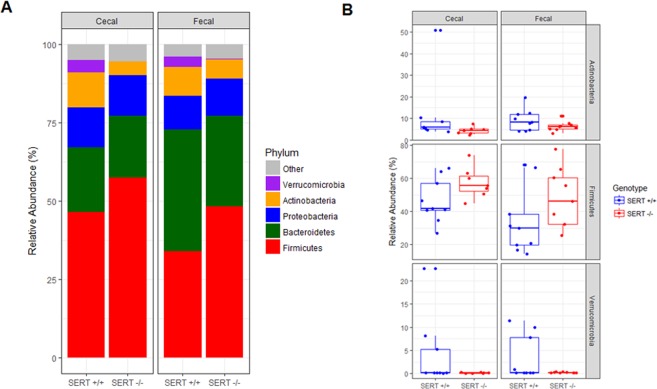
SERT deficiency alters bacterial abundance at the phyla level. (**A**) Bar plot of average abundance of top five phyla in SERT^+/+^ and SERT^−/−^ cecal and fecal samples. (**B**) Box-plots of Firmicutes, Actinobacteria, and Verrucomicrobia, whose abundances differed between SERT^+/+^ and SERT^−/−^ mice. Differential testing by two-way ANOVA, *p* < 0.05 was considered significant.

**Figure 3:**
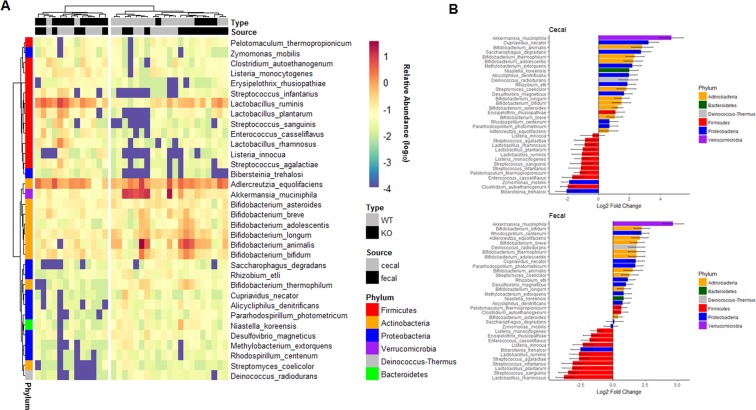
SERT^−/−^ mice have altered abundance of *Akkermansia*, Bacilli, and *Bifidobacterium* species. (**A**) Heat map of differentially abundant species between SERT^+/+^ and SERT^−/−^ mice. Bacterial relative abundances were log_10_ transformed for visualization. Differential abundance testing was performed by likelihood ratio test (*DESeq. 2*), adjusted *p* < 0.1 (Benjamini-Hochberg multiple testing correction) was considered significant. Hierarchal clustering was used to separate samples based on abundances of differentially abundant bacteria. Fisher’s exact test was used to determine if SERT^+/+^ and SERT^−/−^ mice were significantly discriminated by hierarchal clustering, *p* < 0.05 was considered significant. (**B**) Log2 fold changes of species that were differentially abundant.

To determine if SERT deficiency alters bacterial abundance, the top five most abundant bacterial phyla in SERT^−/−^ and SERT^+/+^ mice were compared. The top five phyla accounted for greater than 90% of all reads in every sample (Fig. 2A, Table E2). There was a significant (*p* < 0.05, Two-way ANOVA) increase in the abundance of Firmicutes in SERT^−/−^ compared to SERT^+/+^ mice (Fig. 2B). There were decreases (*p* = 0.10 and *p* < 0.05, Two-way ANOVA) in Actinobacteria and Verrucomicrobia abundances in SERT^−/−^ mice compared to SERT^+/+^ (Fig. 2B). There was a trend (*p* = 0.06, Two-way ANOVA) towards lower Firmicutes in fecal isolates compared to cecal isolates, whereas there was no difference in the abundances of Actinobacteria or Verrucomicrobia between cecal and fecal isolates. To identify changes in species abundances across multiple sampling sites between SERT^+/+^ and SERT^−/−^ mice bacterial counts were assessed as function of two different models. In the full model, bacterial counts were modeled as a function of the strain of mice (SERT^+/+^ vs. SERT^−/−^), the source of sampling (cecal vs. fecal), the batch that the samples were sequenced, and the interaction between strain and sampling. In the reduced model, bacterial counts were modeled only as a function of the source of sampling and the batch that the samples were sequenced. 34 species of bacteria were identified whose variance was significantly estimated (Benjamini-Hochberg corrected *p* < 0.1, Likelihood Ratio Test *DESeq. 2*) by the full model compared to the reduced model (Table E3). In agreement with results seen at the phyla level of taxonomy 22 of 34 differentially abundant species were of the phylum, Firmicutes, Actinobacteria, and Verrucomicrobia (Table E4). Notably, of the species that belonged to other phyla, 10 were Proteobacteria, 1 was Bacteroidetes, and 1 was Deinococcus-Thermus, all of which had very low abundances (median relative abundance <0.05%). Using these 34 differentially abundant bacteria, SERT^+/+^ and SERT^−/−^ mice could be significantly discriminated (*p* = 0.001 accuracy = 80%, Fisher’s Exact test) from each other using hierarchal clustering (Fig. 3A). There were several notable trends with respect to differences between SERT^−/−^ and SERT^+/+^ mice. First, SERT^−/−^ mice displayed higher abundances of species that can be categorized as Bacilli, including those of genera Lactobacillus, Streptococcus, Enterococcus, and Listeria. Of these Bacilli species, *Lactobacillus ruminis* was the most abundant. Secondly, SERT^−/−^ mice have significantly lower abundances of *Bifidobacterium* species and *Akkermansia muciniphilia* compared to SERT^+/+^ mice (Fig. 3B). These results confirm the phylum level observations.

### Bacterial community structure is altered in SERT deficient mice

**Figure 4:**
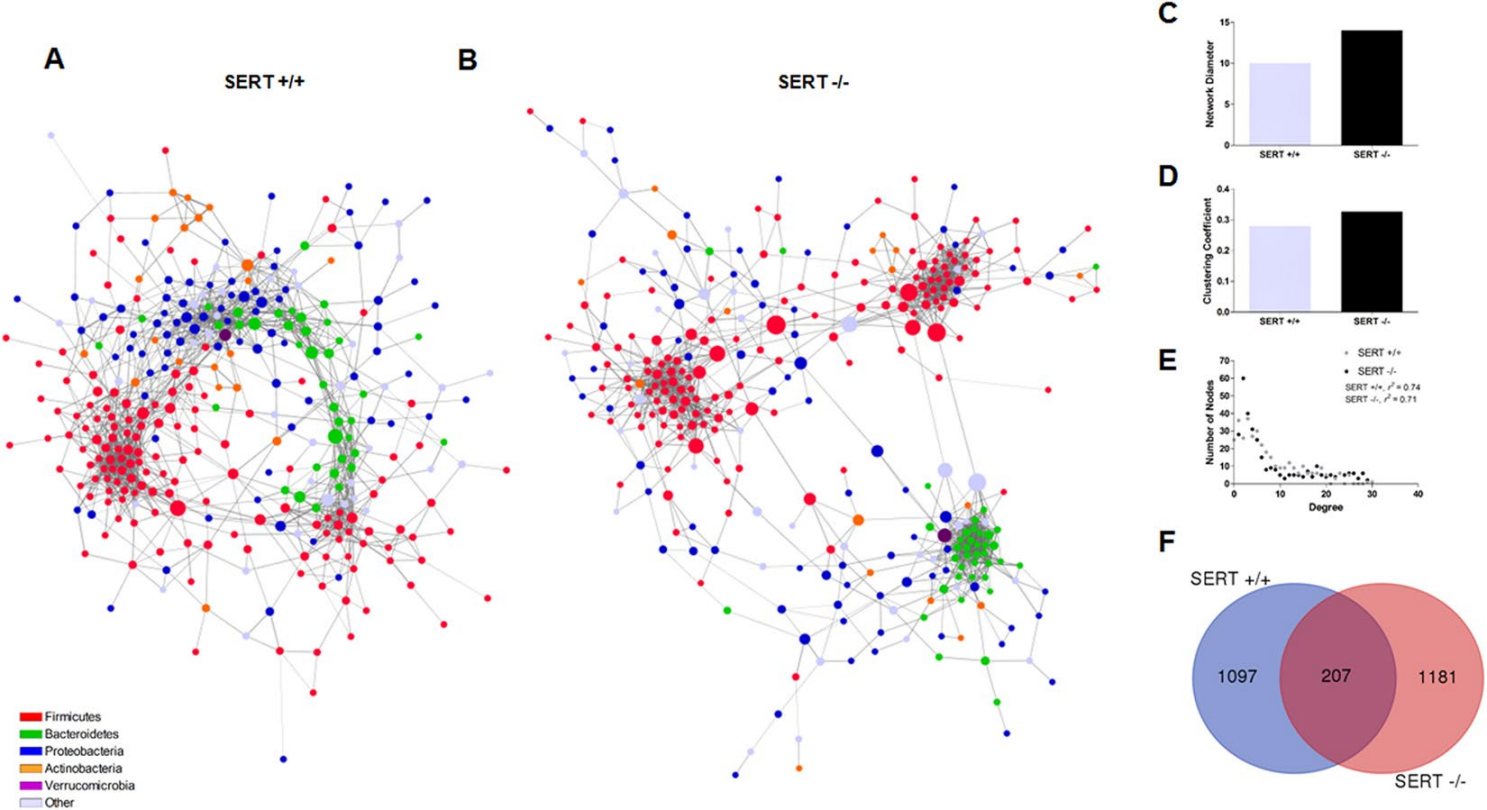
SERT^−/−^ mice have altered bacterial community structure. Bacterial-bacterial co-occurrence networks were generated for significant co-occurrences in (**A**) SERT^+/+^ and (**B**) SERT^−/−^ mice. Bacteria are colored by class and sized is by betweeness centrality. Significant edge testing was determined using SPARCC with 500 bootstraps, *p* < 0.001 was considered significant. Differences in (**C**) network diameter, (**D**) clustering coefficient, (**E**) scale-free fit, and (**F**) overlap in edges display differential network structure.

In the mammalian intestine, bacteria co-exist with one another and are highly dependent on other species to provide specific nutrients required for growth. Due to this, many bacteria exist in specific communities or ecological niches within the gut. To assess community structure differences between SERT^−/−^ and SERT^+/+^ mice, co-occurrence networks were generated for highly significant (*p* < 0.001, *SPARCC*) bacterial species-species co-occurrences (Fig. 4A,B). The networks for SERT^−/−^ and SERT^+/+^ were highly dissimilar. SERT^−/−^ mice co-occurrence network displayed a higher network diameter, clustering coefficient, and had a lower scale-free network fit compared to SERT^+/+^ mice (Fig. 4C–E). Additionally, of the 2485 total co-occurrences observed only 207 (<1%) were observed between both SERT^−/−^ and SERT^+/+^ co-occurrence networks (Fig. 4F). These overlapping co-occurrences between the SERT^−/−^ and SERT^+/+^ revealed three distinct communities dominated by specific phylogenies: Bacteroidetes, Clostridia, and Bacilli dominant communities (Fig. S1A). Notably, the interactions between these communities were dissimilar between SERT^−/−^ and SERT^+/+^. In SERT^+/+^ mice many Bacteroidetes species co-occur with Bacilli species, whereas in SERT^−/−^ there is no co-occurrence between Bacteroidetes species and Bacilli species (Fig. S1B,C). In addition to these conserved communities, it was observed that a community of Bifidobacterium species co-occurred in SERT^+/+^ but was absent SERT^−/−^ (Fig. S1D,E). Changes in community structure could be due to loss or gain of hub species. To assess for hub species in each network, we defined hubs as species with >90^th^ percentile of degree and closeness centrality of their network (Fig. S2). Notably, several *Firmicutes* species were identified as hubs in both networks, whereas *Akkermansia muciniphilia*, several *Proteobacteria*, and *Bacteroidetes* species where only identified as hubs in SERT^+/+^ mice. *Akkermansia muciniphilia* was decreased in SERT^−/−^ mice indicating that loss of a hub species is associated with changes in the community structure of the microbiome in SERT^−/−^ mice.

### Differentially abundant bacteria are associated with gene expression changes observed in SERT deficient mice

**Figure 5:**
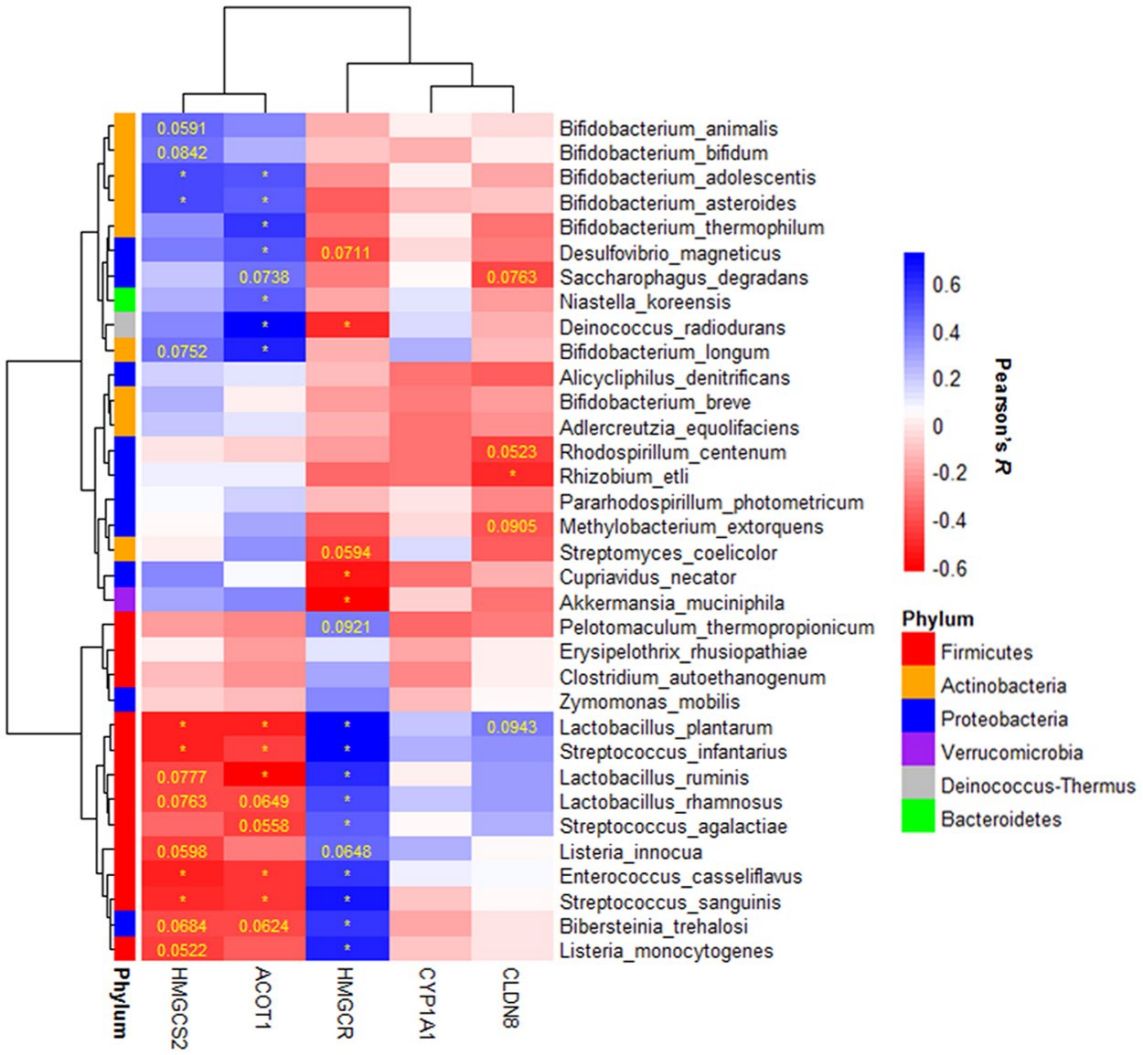
Differentially abundant bacteria significantly correlate with gene expression of HMGCS2 and HMGCR. Heatmap of Pearson’s correlations between *HMGCS2* and *HMGCR* gene expression (ΔCt) and the abundance (log_10_ relative abundance) of differentially abundant bacteria.

SERT^−/−^ mice have been shown to have differential gene expression changes compared to SERT^+/+^ mice (GEO accession GSE93534). To test if changes in the microbiome composition were associated with gene expression changes in SERT^−/−^ mice, the 34 differentially abundant bacteria were correlated with the ΔCt of *Hmgcs2, Cyp1a1, Cldn8*, and *Acot1* in the ileal mucosa and *Hmgcr* in the liver (Fig. 5). It was observed that *Bifidobacterium* species and *Bacilli* species had many significant associations with *Hmgcs2*, *Acot1*, and *Hmgcr*. Neither group of bacteria was highly associated with the expression of *Cyp1a1* or *Cldn8*. This indicates that changes in lipid metabolism that are observed in SERT^−/−^ are associated with changes in the composition of the microbiome.

### SERT deficiency is associated with changes in the abundance of genes involved in fatty acid metabolism

**Figure 6:**
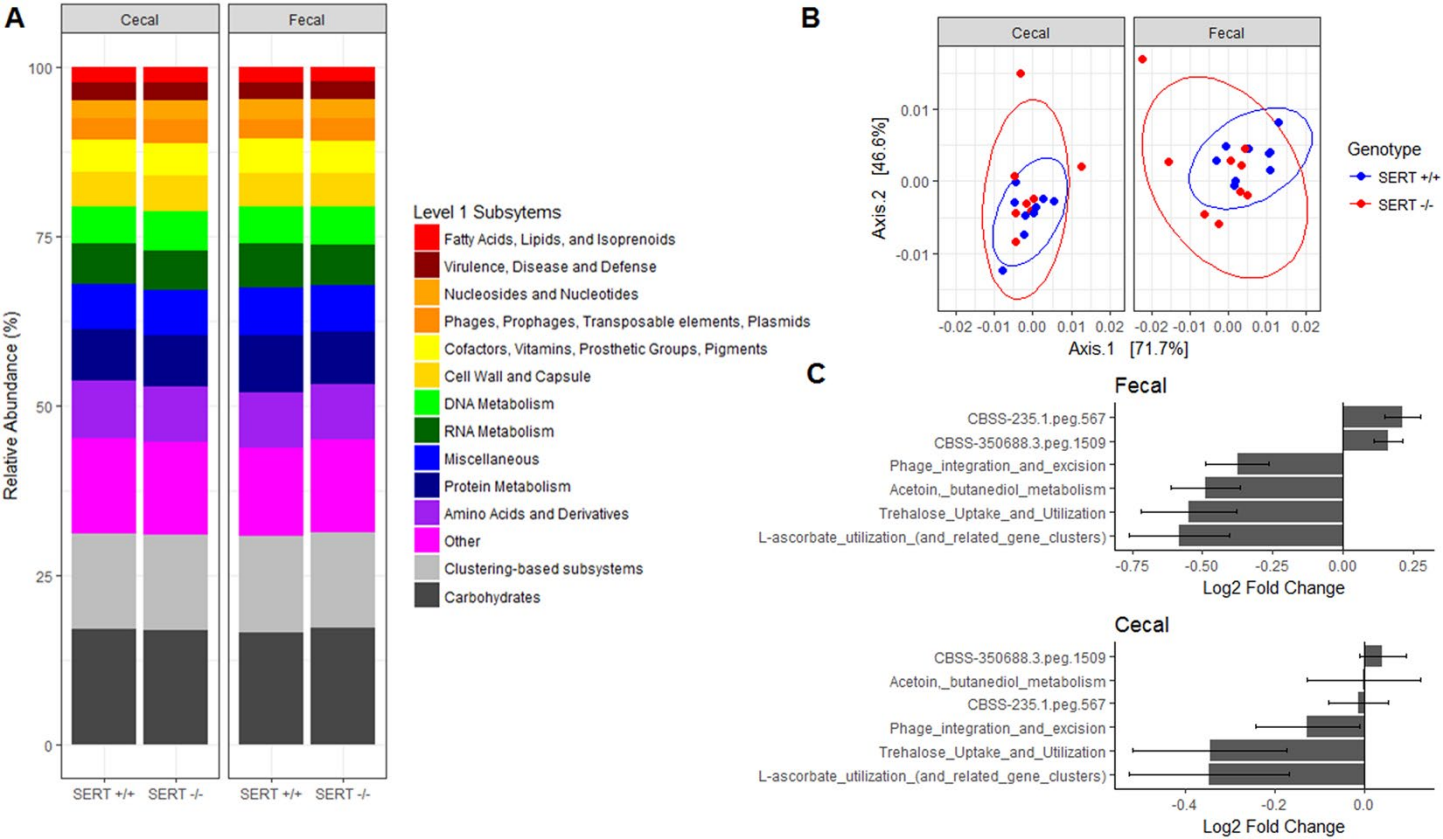
SERT^−/−^ mice are enriched for pathways involved in sugar and l-ascorbate metabolism. (**A**) Bar plot of average abundance of top Level 1 SEED pathways (>2% abundance) in SERT^+/+^ and SERT^−/−^ cecal and fecal samples. (**B**) PCoA of Level 3 SEED pathways, displaying the first two principle components, Jensen-Shannon divergence between SERT^+/+^ and SERT^−/−^ cecal and fecal samples. Differential testing PERMANOVA using *adonis*, *p* < 0.05. (**C**) Log2 fold changes of pathways that differentially abundant. Differential abundance testing was performed by likelihood ratio test (*DESeq. 2*), adjusted *p* < 0.1 (Benjamini-Hochberg multiple testing correction) was considered significant.

**Figure 7:**
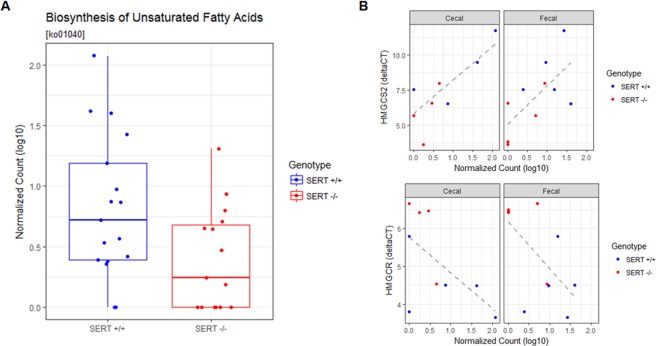
Abundance of genes involved in the biosynthesis of unsaturated fatty acids are depleted in SERT^−/−^ mice and are associated with the expression of HMGCS2 and HMGCR. (**A**) Abundance (log_10_ normalized count) of the KEGG pathway, biosynthesis of unsaturated fatty acids [ko01040]. Differential abundance testing was performed by likelihood ratio test (*DESeq. 2*), adjusted *p* < 0.1 (Benjamini-Hochberg multiple testing correction) was considered significant. (**B**) Linear regression between gene expression of HMGCS2 (ΔCt) and abundance of the KEGG pathway, biosynthesis of unsaturated fatty acids [ko01040], *p* < 0.05 was considered significant. Points are colored by SERT^+/+^ and SERT^−/−^ mice.

**Figure 8:**
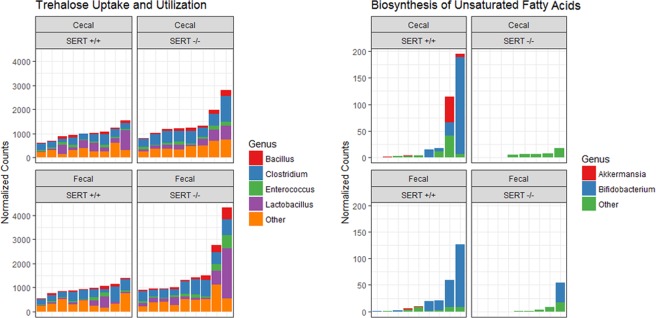
Differentially abundant bacteria are sources for differentially abundant functional pathways. Two representative pathways are shown as stacked bar graphs, where the contribution of each genus to the total reads assigned to a pathway is displayed.

To assess if there were any changes in the abundance of functional categories of genes, SEED and KEGG pathway counts were assessed. Broad categorizations of metabolic pathways showed no differences (Fig. 6A). To test if the composition of functions was significantly different, we compared level 3 SEED pathways using PCoA (Fig. 6B). Functional compositions were significantly different (PERMANOVA, *p* < 0.05) between strain of mice (SERT^+/+^ vs. SERT^−/−^), the source of sampling (cecal vs. fecal), but not between batch. In the full model, SEED or KEGG pathways were modeled as a function of the strain of mice (SERT^+/+^ vs. SERT^−/−^), the source of sampling (cecal vs. fecal), the batch that the samples were sequenced, and the interaction between strain and sampling. In the reduced model, SEED or KEGG pathway counts were modeled only as a function of the source of sampling and the batch that the samples were sequenced. Using SEED level 3 classifications, we found three pathways that were significantly enriched (Benjamini-Hochberg corrected *p* < 0.1, Likelihood Ratio Test *DESeq. 2*) in SERT^+/+^ in both fecal and cecal samples: l-ascorbate utilization, trehalose uptake and utilization, and phage integration and excision (Table E5). We identified a single KEGG pathway, biosynthesis of unsaturated fatty acids [ko01040], whose variance was significantly estimated (Benjamini-Hochberg corrected *p* < 0.1, Likelihood Ratio Test *DESeq. 2*) by the full model compared to the reduced model (Fig. 7A, Table E6). This pathway was significantly more abundant in the SERT^+/+^ mice compared to SERT^−/−^ mice (Fig. 7A). This pathway also significantly associated (*p* < 0.05, Pearson’s correlation) with the expression of both *Hmgcs2* and *Hmgcr* (Fig. 7B). This pathway did not associate with *Acot1, Cyp1a1*, or *Cldn8* (data not shown). The main contributors to these pathways were notably differentially abundant bacteria, *Firmicutes*, *Bifidobacterium*, and *Akkermansia* (Fig. 8). This indicates that loss and gains of species are not completely functionally redundant and result in changes in functional capabilities of the microbiome.

## Discussion

The gut microbiome plays an important role in the pathogenesis of various health disorders^32–36^. In the current study, the fecal and cecal microbiomes of adult SERT^+/+^ and SERT^−/−^ mice were sequenced and analyzed under basal conditions. Fecal microbiota likely represent a mixture of indigenous and transient microbes from the entire GI tract^37^. We demonstrate that the loss of SERT leads to alterations in gut microbial composition, which is consistent with the metabolic syndrome observed in these mice. Interestingly, changes in metabolic capabilities of the gut microbiome were associated with changes in host gene expression of enzymes involved in lipid metabolism in SERT^−/−^ mice. Collectively, our study confirms that the loss of SERT in mice is associated with gut microbial dysbiosis and this dysbiosis correlates with metabolic alterations within the host.

In our study, β-diversity showed a significant difference between SERT^+/+^ and SERT^−/−^ mice. SERT^−/−^ mice had less variance in gut microbial compositions, indicating that SERT deficiency is exerting a selective pressure on the gut microbiome. On the taxonomic level, the SERT^−/−^ mice had a significant increase in Firmicutes as compared to SERT^+/+^ mice. Previous studies have shown that the abundance of Firmicutes is associated with obesity in mice^38,39^. While the exact mechanism for this association is not certain, it is thought that Firmicutes have a high capacity to hydrolyze non-digestible polysaccharides into molecules which are absorbed by the host and stored as excess calories^40^. We observed increased abundance of genes involved in trehalose, a disaccharide, uptake and utilization in SERT^−/−^ mice. Interestingly, it was recently shown that epidemic ribotypes of *Clostridium difficile* have acquired mutations in trehalose metabolizing enzymes which increases their virulence^41^. Studies in zebrafish have shown that higher abundance of Firmicutes leads to increase in the number and sizes of the lipid droplets thus promoting fatty acid absorption in the extra intestinal tissues^42^. Among the differentially abundant Firmincutes, *Lactobacillus ruminis* was the most abundant. *L. ruminis* is a lactic acid producing bacteria that is known to exhibit commensal relationship with its host^43^. Interestingly, the abundance of *L. ruminis* has been observed at higher abundance in overweight and obese patients^44^.

Actinobacteria and Verrucomicrobia, as well as *Bifidobacteria*, showed decreased abundances in SERT^−/−^ mice as compared to SERT^+/+^ mice. *Bifidobacteria*, are one of the abundant probiotic species present in the mammalian gut^45^. Previous studies in rats have shown anti-obesity and lipid lowering effects of these bacteria when supplemented along with a high-fat diet and an inverse correlation with BMI^46,47^. Thus, low abundance of *Bifidobacteria* species including *B. bifidum*, *B. animalis*, and *B. breve* could be associated with the metabolic disturbances observed in SERT^−/−^ mice. *Akkermansia muciniphila* of the phylum Verrucomicrobia was significantly less abundant in SERT^−/−^ mice as compared to SERT^+/+^ mice. Many animal models of metabolic syndrome had lower levels of *A. muciniphila*^48^. Clinical studies show an inverse correlation of *A. muciniphila* to metabolic parameters including fasting glucose levels, waist-to-hip ratio, and subcutaneous adipocyte diameter^48,49^. Further, interventional studies in mouse models have provided evidence of a direct role of *A. muciniphila* in reversing metabolic disorders^50,51^.

Microarray analysis of the small intestinal mucosa revealed remarkable differences in gene expression in SERT^−/−^ mice as compared to SERT^+/+^ mice^31^. Of interest, several genes involved in lipid metabolism were up-regulated including *Hmgcs2*, *Bdh2*, *Acot1*, and *Acot2*. *Hmgcr* was found to be down-regulated in the liver of SERT^−/−^ mice, as expected due to the hepatic steatosis that develops in SERT^−/−^ mice^22^. Interestingly, gut microbiota can have profound systemic effects^52^. Notably, in our studies both the cecal and fecal microbiota changes correlated with gene expression changes related to lipid metabolism not only in ileum but also in the liver. For example, our analysis showed that among the 34 differentially abundant bacteria, the expression of intestinal *Hmgcs2*, *Acot1* and liver *Hmgcr* associated significantly with the abundance of several *Bifidobacterium* species and *Bacilli* species.

The gut microbial community performs a range of useful functions for the host, including digesting substrates inaccessible to host enzymes, educating the immune system, and repressing the growth of harmful microorganisms. We identified several pathways that are associated with SERT deficiency. One pathway, biosynthesis of unsaturated fatty acids [ko01040], was significantly less abundant in SERT^−/−^ mice as compared to SERT^+/+^. The most abundant gene in this pathway was *tesB*, which encodes a bacterial acyl-CoA thioesterase (*Acot*). *tesB* was nearly absent in SERT^−/−^ mice, in contrast to the dramatic upregulation of host *Acot* enzymes which catalyze the same biochemical reactions. This implies that the host and microbiome share redundant functions. From our analysis, it is not clear whether the host selects for microbial function or vice versa.

The community structure of the microbiome was significantly altered in SERT^−/−^ mice as compared to SERT^+/+^ mice. The SERT^−/−^ microbiome consisted of disconnected sub-networks, in contrast to SERT^+/+^ where microbial networks were more cohesive. This indicates loss of interaction between bacteria in SERT^−/−^. In addition, the number of overlapping co-occurrences seen in SERT^+/+^ and SERT^−/−^ were drastically decreased. There was also a loss of several hub species, those species which have large influence over the network, in SERT^−/−^ mice which also had decreased abundance in SERT^−/−^, *A. muciniphila* and *Desulfovibrio*. It is possible loss a hub species, such as *A. muciniphila* and *Desulfovibrio*, could change the abundance of many other species due to their large influence on the network. SERT deficiency could therefore, dramatically alter the stability of bacterial communities, thus disturbing bacterial homeostasis.

There are a few limitations to our study. First, it is not clear whether changes in metabolic capacity of the microbiome are causing changes in host metabolic pathways in SERT^−/−^ mice, or vice-versa. Our study design did not allow for examination of temporal relationships which could allow for casual determinations. A recent study has characterized the fecal microbiome in SERT^+/+^ and SERT^−/−^ rats, and the compounding effect of genotype and maternal separation^53^. Similar to SERT^−/−^ mice, SERT^−/−^ rats also exhibit a metabolic syndrome-like phenotype which has been characterized at 12 weeks of age^54^. However, the rat microbiome study was performed at 3 weeks of age, therefore any microbiome changes related to metabolic syndrome may be absent. Nonetheless, there were similarities to our current study (e.g., both the rat study and our study showed an increase in the prevalence of Firmicutes and reduced *Desulfovibrio* spp). In contrast to our findings, there was an increase in Fusobacteria in the rat study. They also did not report any changes in bacteria that were drastically altered in our study such as *A. muciniphila* or *Bifidobacteria*. In mice, previous studies have shown that 3-month old SERT^−/−^ mice do not exhibit an increase in body weight and body fat content, although they did manifest glucose and insulin intolerance^22^. These findings suggested that the metabolic deficits developed prior to a significant increase in the adiposity (around 6 months) in SERT^−/−^ mice via estrogen dependent pathways. Future studies should focus on the temporal analysis of microbiota both in SERT^−/−^ and SERT over-expressing mice to provide insights into age dependent effects of SERT on obesity.

Collectively, our data indicates that SERT plays a vital role in maintaining homeostasis of the gut microbiota, and that its deficiency leads to loss of bacterial niches and altered microbial metabolic capabilities. This is clinically relevant, as selective serotonin reuptake inhibitors (SSRIs) are commonly prescribed for treatment of depression and other psychiatric disorders. Additionally, SERT expression is decreased in several pathophysiological conditions, including inflammatory bowel diseases and irritable bowel syndrome. Thus, understanding the contribution of SERT to gut microbial homeostasis is crucial. Overall, the findings of the current study establish a strong foundation for understanding the involvement of the intestinal microbiome in the pleotropic consequences of SERT deficiency.

## Methods

### Mice

SERT^+/+^ and SERT^−/−^ mice of C57BL/6 background (purchased from Jackson Laboratory) were bred in-house at the Jesse Brown Veterans Affairs Medical Center animal facility. Animals were housed in autoclaved polypropylene cages with corncob bedding under a specific pathogen-free environment under a 12 h light/dark cycle. Genotyping was carried out after tail clipping on 4 weeks old mice. The mice were co-housed by genotype until 11–12 weeks of age when fecal and cecum stool samples were collected. Two separate batches of mice consisting of both males and females were used for the current study. All animal studies were performed in accordance with institutional guidelines and regulations and as approved by the Animal Care Committees of the University of Illinois at Chicago and the Jesse Brown Veterans Affairs Medical Center.

### Microbial Sampling and DNA Isolation

Mice were placed on wire bottom cages for 24 h to collect fecal pellets and stored at −80 °C. Following this, mice were anesthetized and dissected. Fresh cecal content was collected from the gut and placed into autoclaved microcentrifuge tubes, and stored at −80 °C till further use. One or two fecal pellets, and 25–50 mg of cecal content was used for metagenomic DNA isolation using PowerSoil DNA isolation kit (Catalogue # 12888-100, MO BIO Laboratories, Inc) using the method as described previously^55^. The quality and quantity of the DNA was accessed using a spectrophotometer (NanoPhotometer Pearl, Denville Scientific, Inc), agarose gel electrophoresis, and fluorometer (Qubit® dsDNA High Sensitivity and dsDNA Broad Range assay, Life Technologies Corporation).

### Metagenomic Library Preparation and Metagenomic Shotgun Sequencing

To prepare Illumina sequencing compatible libraries for shotgun sequencing, 500 ng metagenomic DNA was mechanically sheared to generate 450–600 bp fragments using a Covaris S220 instrument (Covaris, Inc). Fragmented metagenomic DNA was end-repaired and 3′-adenylated, ligated with Illumina adapters, and PCR enriched with Illumina sequencing indexes (barcodes) using the NEBNext Ultra DNA library prep kit for Illumina (Catalogue # E7370L,New England BioLabs Inc). The quality and quantity of all the DNA libraries were analyzed with an Agilent DNA 1000 Kit on the 2100 Bioanalyzer Instrument and Qubit. DNA libraries were pooled in equimolar concentration and were sequenced following manufacturer’s protocol by multiplexing on Illumina MiSeq using the v3-600 kit for 301 paired-end read length and included an additional 12 cycles for the index. The average sequencing depth was approximately 3 million paired end reads per sample or 1.03 Gbp per sample.

### Taxonomic and Functional Profiling

The produced metagenomic sequencing reads were processed with our custom data analysis pipeline “WE VOTE” that is hosted on the UIC supercomputer “Extreme”. First, the sequences were quality controlled by filtering out all low-quality reads (<25 on Phred quality score), short reads (<100 bp), or any mouse reads. High-quality microbial reads were then assembled into longer contigs using MetaVelvet^56^. For each sample, the microbial taxonomic profile was constructed using WEVOTE^57^. Since WEVOTE is an ensemble classifier, we used Kraken, Clark, and BLASTN as base classifiers for WEVOTE^58,59^. Using this approach, we were able to classify an average of 230,610 reads per sample at the species level. To identify specific functions in the microbiome, reads were upload to MG-RAST and annotated using the KEGG and SEED databases with default parameter settings^60^. Functions were assessed at the pathway level 3.

### RNA Extraction and RT-qPCR

Total RNA was extracted from mouse ileal mucosal scrapings or liver using Qiagen RNeasy kits. RNA was reverse transcribed and amplified using a Brilliant SYBR Green qRT-PCR Master Mix kit (Stratagene). The gene-specific primer sequences are listed in Supplementary Table E1. The relative mRNA levels were normalized to *Gapdh* mRNA levels using the ΔΔCt method.

### Statistics

All statistical analyses were performed in *R* (Version 3.3) unless otherwise specified. Diversity metrics, Shannon Index and Jensen-Shannon divergence, were calculated using *phyloseq*.^61^. Shannon index was compared by two-way ANOVA. Jensen-Shannon divergence was compared by permutational ANOVA (PERMANOVA) modeling for mouse genotype, source, and batch of metagenome. For phylum level analysis, species counts were agglomerated and assess using two-way ANOVA. *P* < 0.05 was considered statistically significant. Differentially abundant species and KEGG pathways were identified using likelihood ratio test in DESeq. 2^62^. Counts were modeled as a function of mouse genotype (SERT^+/+^ vs. SERT^−/−^), source (Fecal vs. Cecal), the interaction between source and genotype, and batch in the full model and source and batch in the reduced model. Benajmini-Hochberg corrected for multiple testing *P* < 0.1 was considered statistically significant. To test for associations between species abundance and gene expression, normalized counts (DESeq. 2 median sum scaling) of species were correlated with ΔCt of genes of interest. Pearson’s correlation coefficient was used to assess statistical significance, *P* < 0.05 was considered statistically significant. ΔCt represents reduced gene expression therefore correlation coefficient is opposite of the effect. Co-occurrence networks were constructed using *SPARCC* with bootstrapping (*B* = 500) in the *spieceasi* package^63^. Edges with *P* < 0.001 and positive correlations were considered significant edges (ie significant co-occurrences). Network graphs and statistics were plotted and calculated in *Cytoscape*^64^.

### Study Approval

Animal studies were approved by the Animal Care Committees of the University of Illinois at Chicago and the Jesse Brown Veterans Affairs Medical Center. All studies were conducted in accordance with institutional guidelines and regulations at the University of Illinois at Chicago and the Jesse Brown Veterans Affairs Medical Center.

## Supplementary information

Figure S1, Figure S2, Table E1, Table E2

Table E3

Table E4

Table E5

Table E6

## References

[1] Chen JJ (2001). Maintenance of serotonin in the intestinal mucosa and ganglia of mice that lack the high-affinity serotonin transporter: Abnormal intestinal motility and the expression of cation transporters. J. Neurosci..

[2] Hansen MB, Witte A-B (2008). The role of serotonin in intestinal luminal sensing and secretion. Acta Physiol (Oxf).

[3] Hansen MB, Skadhauge E (1997). Signal transduction pathways for serotonin as an intestinal secretagogue. Comp. Biochem. Physiol. A Physiol..

[4] Mawe GM, Hoffman JM (2013). Serotonin signalling in the gut—functions, dysfunctions and therapeutic targets. Nat Rev Gastroenterol Hepatol.

[5] Gill RK (2008). Serotonin modifies cytoskeleton and brush-border membrane architecture in human intestinal epithelial cells. AJP: Gastrointestinal and Liver Physiology.

[6] Saksena S (2005). Involvement of c-Src and protein kinase C delta in the inhibition of Cl(-)/OH- exchange activity in Caco-2 cells by serotonin. J. Biol. Chem..

[7] Gill RK (2005). Serotonin inhibits Na+/ H +exchange activity via 5-HT4 receptors and activation of PKC alpha in human intestinal epithelial cells. Gastroenterology.

[8] Vieira-Coelho MA, Teixeira VL, Guimarães JT, Serrão MP, Soares-da-Silva P (1999). Caco-2 cells in culture synthesize and degrade dopamine and 5-hydroxytryptamine: a comparison with rat jejunal epithelial cells. Life Sci..

[9] Martel F, Monteiro R, Lemos C (2003). Uptake of serotonin at the apical and basolateral membranes of human intestinal epithelial (Caco-2) cells occurs through the neuronal serotonin transporter (SERT). J. Pharmacol. Exp. Ther..

[10] Yadav VK (2008). Lrp5 controls bone formation by inhibiting serotonin synthesis in the duodenum. Cell.

[11] Ziu E (2012). Down-regulation of the serotonin transporter in hyperreactive platelets counteracts the pro-thrombotic effect of serotonin. J. Mol. Cell. Cardiol..

[12] Sumara G, Sumara O, Kim JK, Karsenty G (2012). Gut-derived serotonin is a multifunctional determinant to fasting adaptation. Cell Metab..

[13] Kéreveur A (2000). High plasma serotonin levels in primary pulmonary hypertension. Effect of long-term epoprostenol (prostacyclin) therapy. Arterioscler. Thromb. Vasc. Biol..

[14] Bengel D (1998). Altered brain serotonin homeostasis and locomotor insensitivity to 3, 4-methylenedioxymethamphetamine (‘Ecstasy’) in serotonin transporter-deficient mice. Mol. Pharmacol..

[15] Kim D-K (2005). Altered serotonin synthesis, turnover and dynamic regulation in multiple brain regions of mice lacking the serotonin transporter. Neuropharmacology.

[16] Epperson CN, Jatlow PI, Czarkowski K, Anderson GM (2003). Maternal fluoxetine treatment in the postpartum period: effects on platelet serotonin and plasma drug levels in breastfeeding mother-infant pairs. Pediatrics.

[17] Tjurmina OA, Armando I, Saavedra JM, Goldstein DS, Murphy DL (2002). Exaggerated adrenomedullary response to immobilization in mice with targeted disruption of the serotonin transporter gene. Endocrinology.

[18] Schmitt A (2003). Organic cation transporter capable of transporting serotonin is up-regulated in serotonin transporter-deficient mice. J. Neurosci. Res..

[19] Murphy DL, Lesch K-P (2008). Targeting the murine serotonin transporter: insights into human neurobiology. Nat. Rev. Neurosci..

[20] Warden SJ, Haney EM (2008). Skeletal effects of serotonin (5-hydroxytryptamine) transporter inhibition: evidence from *in vitro* and animal-based studies. J Musculoskelet Neuronal Interact.

[21] Zha W, Ho HTB, Hu T, Hebert MF, Wang J (2017). Serotonin transporter deficiency drives estrogen-dependent obesity and glucose intolerance. Sci Rep.

[22] Chen X, Margolis KJ, Gershon MD, Schwartz GJ, Sze JY (2012). Reduced serotonin reuptake transporter (SERT) function causes insulin resistance and hepatic steatosis independent of food intake. PLoS ONE.

[23] Medici V, McClave SA, Miller KR (2016). Common Medications Which Lead to Unintended Alterations in Weight Gain or Organ Lipotoxicity. Curr Gastroenterol Rep.

[24] Gomez de Agüero M (2016). The maternal microbiota drives early postnatal innate immune development. Science.

[25] Chou, C. J., Membrez, M. & Blancher, F. Gut decontamination with norfloxacin and ampicillin enhances insulin sensitivity in mice. *Nestle Nutr Workshop Ser Pediatr Program* **62**, 127–37– discussion 137–40 (2008). 10.1159/000146256 18626197

[26] Festi D (2014). Gut microbiota and metabolic syndrome. World J. Gastroenterol..

[27] Reigstad CS (2015). Gut microbes promote colonic serotonin production through an effect of short-chain fatty acids on enterochromaffin cells. FASEB J..

[28] Yano JM (2015). Indigenous bacteria from the gut microbiota regulate host serotonin biosynthesis. Cell.

[29] Singhal M (2017). Role of SHP2 protein tyrosine phosphatase in SERT inhibition by enteropathogenic E. coli (EPEC). Am. J. Physiol. Gastrointest. Liver Physiol..

[30] Esmaili A (2009). Enteropathogenic Escherichia coli infection inhibits intestinal serotonin transporter function and expression. Gastroenterology.

[31] Manzella C (2018). Serotonin is an endogenous regulator of intestinal CYP1A1 via AhR. Sci Rep.

[32] Casén C (2015). Deviations in human gut microbiota: a novel diagnostic test for determining dysbiosis in patients with IBS or IBD. Aliment. Pharmacol. Ther..

[33] Youmans BP (2015). Characterization of the human gut microbiome during travelers’ diarrhea. Gut Microbes.

[34] Jones-Hall YL, Nakatsu CH (2016). The Intersection of TNF, IBD and the Microbiome. Gut Microbes.

[35] Maruvada P, Leone V, Kaplan LM, Chang EB (2017). The Human Microbiome and Obesity: Moving beyond Associations. Cell Host Microbe.

[36] Soverini M (2017). Variation of Carbohydrate-Active Enzyme Patterns in the Gut Microbiota of Italian Healthy Subjects and Type 2 Diabetes Patients. Front Microbiol.

[37] Hillman ET, Lu H, Yao T, Nakatsu CH (2017). Microbial Ecology along the Gastrointestinal Tract. Microbes Environ..

[38] Turnbaugh PJ (2006). An obesity-associated gut microbiome with increased capacity for energy harvest. Nature.

[39] Boulangé CL, Neves AL, Chilloux J, Nicholson JK, Dumas M-E (2016). Impact of the gut microbiota on inflammation, obesity, and metabolic disease. Genome Med.

[40] Chakraborti CK (2015). New-found link between microbiota and obesity. World J Gastrointest Pathophysiol.

[41] Collins J (2018). Dietary trehalose enhances virulence of epidemic Clostridium difficile. Nature.

[42] Semova I (2012). Microbiota regulate intestinal absorption and metabolism of fatty acids in the zebrafish. Cell Host Microbe.

[43] Yu X (2015). Human Gut-Commensalic Lactobacillus ruminis ATCC 25644 Displays Sortase-Assembled Surface Piliation: Phenotypic Characterization of Its Fimbrial Operon through In Silico Predictive Analysis and Recombinant Expression in Lactococcus lactis. Plos One.

[44] Yamashiro K (2017). Gut dysbiosis is associated with metabolism and systemic inflammation in patients with ischemic stroke. Plos One.

[45] Milani C (2015). Bifidobacteria exhibit social behavior through carbohydrate resource sharing in the gut. Sci Rep.

[46] An HM (2011). Antiobesity and lipid-lowering effects of Bifidobacterium spp. in high fat diet-induced obese rats. Lipids Health Dis.

[47] Million M (2013). Correlation between body mass index and gut concentrations of Lactobacillus reuteri, Bifidobacterium animalis, Methanobrevibacter smithii and Escherichia coli. Int J Obes (Lond).

[48] Derrien, M., Belzer, C. & de Vos, W. M. Akkermansia muciniphila and its role in regulating host functions. *Microb. Pathog*, 10.1016/j.micpath.2016.02.005 (2016). 10.1016/j.micpath.2016.02.005 26875998

[49] Dao MC (2016). Akkermansia muciniphila and improved metabolic health during a dietary intervention in obesity: relationship with gut microbiome richness and ecology. Gut.

[50] Everard A (2013). Cross-talk between Akkermansia muciniphila and intestinal epithelium controls diet-induced obesity. Proc. Natl. Acad. Sci. U.S.A..

[51] Org E (2015). Genetic and environmental control of host-gut microbiota interactions. Genome Res..

[52] Ho JTK, Chan GCF, Li JCB (2015). Systemic effects of gut microbiota and its relationship with disease and modulation. BMC Immunol..

[53] El Aidy S (2017). Serotonin Transporter Genotype Modulates the Gut Microbiota Composition in Young Rats, an Effect Augmented by Early Life Stress. Front Cell Neurosci.

[54] Homberg JR, la Fleur SE, Cuppen E (2009). Serotonin Transporter Deficiency Increases Abdominal Fat in Female, but Not Male Rats. Obesity.

[55] Ranjan R, Rani A, Metwally A, McGee HS, Perkins DL (2016). Analysis of the microbiome: Advantages of whole genome shotgun versus 16S amplicon sequencing. Biochem. Biophys. Res. Commun..

[56] Namiki T, Hachiya T, Tanaka H, Sakakibara Y (2012). MetaVelvet: an extension of Velvet assembler to de novo metagenome assembly from short sequence reads. Nucleic Acids Res..

[57] Metwally AA, Dai Y, Finn PW, Perkins DL (2016). WEVOTE: Weighted Voting Taxonomic Identification Method of Microbial Sequences. Plos One.

[58] Altschul SF, Gish W, Miller W, Myers EW, Lipman DJ (1990). Basic local alignment search tool. J. Mol. Biol..

[59] Ounit R, Wanamaker S, Close TJ, Lonardi S (2015). CLARK: fast and accurate classification of metagenomic and genomic sequences using discriminative k-mers. BMC Genomics.

[60] Meyer F (2008). The metagenomics RAST server - a public resource for the automatic phylogenetic and functional analysis of metagenomes. BMC Bioinformatics.

[61] McMurdie PJ, Holmes S (2013). phyloseq: an R package for reproducible interactive analysis and graphics of microbiome census data. PLoS ONE.

[62] Love MI, Huber W, Anders S (2014). Moderated estimation of fold change and dispersion for RNA-seq data with DESeq. 2. Genome Biol..

[63] Kurtz ZD (2015). Sparse and compositionally robust inference of microbial ecological networks. PLoS Comput. Biol..

[64] Shannon P (2003). Cytoscape: a software environment for integrated models of biomolecular interaction networks. Genome Res..

