# Supplementary material for: Serotonin Transporter Deficiency is Associated with Dysbiosis and Changes in Metabolic Function of the Mouse Intestinal Microbiome
Source: Sci Rep. 2019 Feb 14;9:2138. doi: 10.1038/s41598-019-38489-8 (PMC6375953; doi:10.1038/s41598-019-38489-8)
Supplement: Supplementary file 1 — Figure S1, Figure S2, Table E1, Table E2 [file 41598_2019_38489_MOESM1_ESM.pdf]

Supplementary Information

Serotonin Transporter Deficiency is Associated with Dysbiosis and Changes in Metabolic Function of the Mouse Intestinal Microbiome

Megha Singhal[1,†], Benjamin A Turturice[† 2,3], Christopher R Manzella[4], Ravi Ranjan[2], Ahmed A Metwally[2, 6], Juliana Theorell[2], Yue Huang[2], Waddah A Alrefai[1,8], Pradeep K Dudeja[1,8], Patricia W Finn[* 2], David L Perkins[* 5, 6, 7], Ravinder K Gill[*,1]

1. Division of Gastroenterology & Hepatology,
2. Division of Pulmonary, Critical Care, Sleep and Allergy,
3. Department of Microbiology & Immunology,
4. Department of Physiology and Biophysics,
5. Division of Nephrology
6. Department of Surgery,
7. Department of Bioengineering,
University of Illinois at Chicago; and
8. Jesse Brown VA Medical Center, Chicago, IL
† Equal contribution
* Co-senior authors (Equal contribution)

Correspondence:
Ravinder K. Gill, Ph.D.
Associate Professor
Division of Gastroenterology & Hepatology
820 South Damen Avenue Chicago, IL 60612
Tel. (312)-569-7463
Fax (312)-569-8114
E-mail: rgill@uic.edu

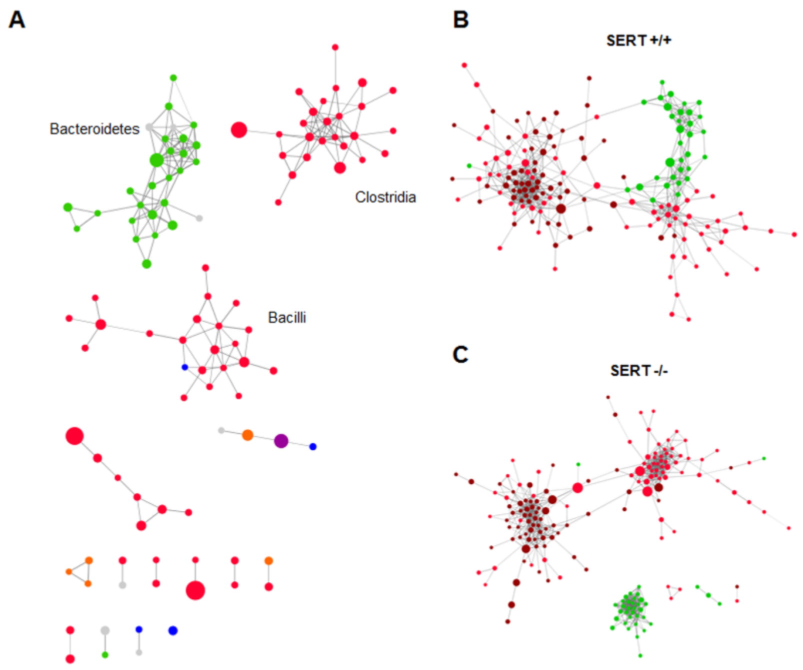

Figure S1. Structural similarities and differences between SERT$^{+/+}$ and SERT$^{-/-}$ co-occurrence networks. (**A)** Edge overlaps between SERT$^{+/+}$ and SERT$^{-/-}$ mice. Three main communities that were shared between SERT$^{+/+}$ and SERT$^{-/-}$ are labelled: Bacteroidetes, Clostridia, and Bacilli. Nodes are colored by their respective phyla: Bacteroidetes (green), Firmicutes (red), Proteobacteria (blue), Actinobacteria (orange), Verrucomicrobia (purple), and Other (grey). Communities of Clostridia (dark brown), Bacilli (red), and Bacteroidetes (green) shown for **(B)** SERT$^{+/+}$ and **(C)** SERT$^{-/-}$ mice. Bifidobacterium (orange) communities and their first neighbors (grey) shown for **(D)** SERT$^{+/+}$ and **(E)** SERT$^{-/-}$ mice.

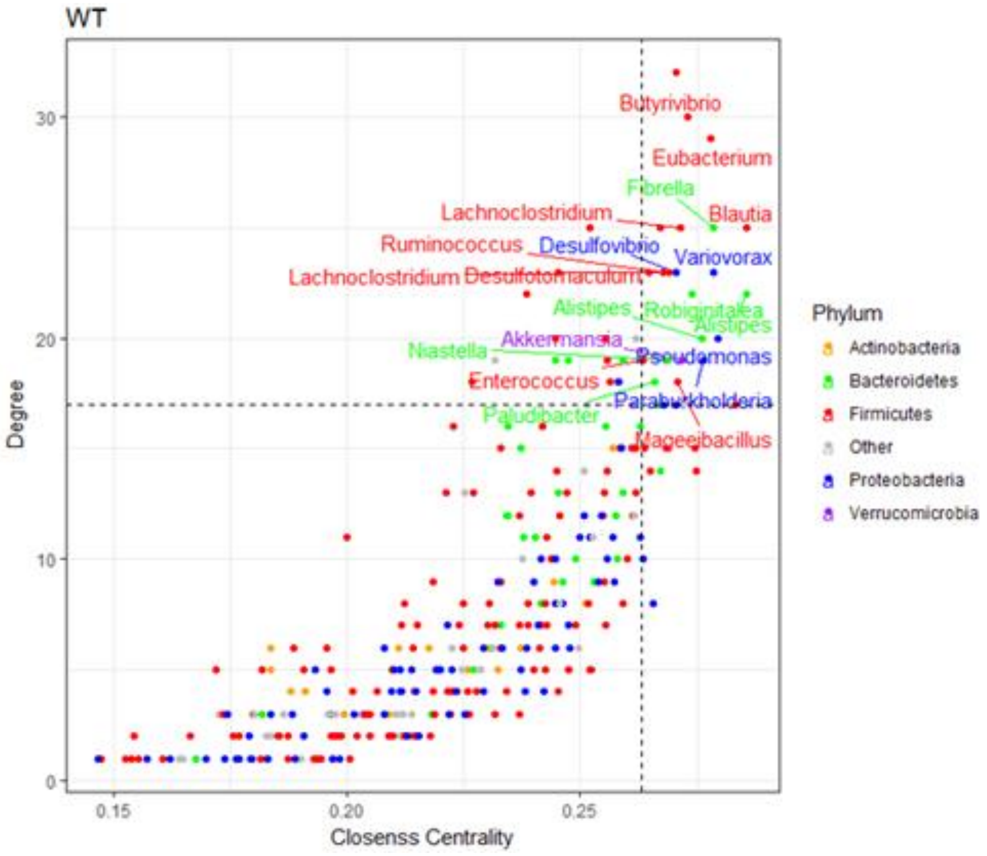
WT
Butyrivibrio
Eubacterium
Fibrella
Blautia
Lachnoclostridium
Ruminococcus
Desulfovibrio
Variovorax
Lachnoclostridium
Desulfotomaculum
Alistipes
Robiginitalea
Alistipes
Niastella
Akkermansia
Pseudomonas
Enterococcus
Paraburkholderia
Paludibacter
Mageibacillus
Degree
Closenss Centrality
Phylum
Actinobacteria
Bacteroidetes
Firmicutes
Other
Proteobacteria
Verrucomicrobia

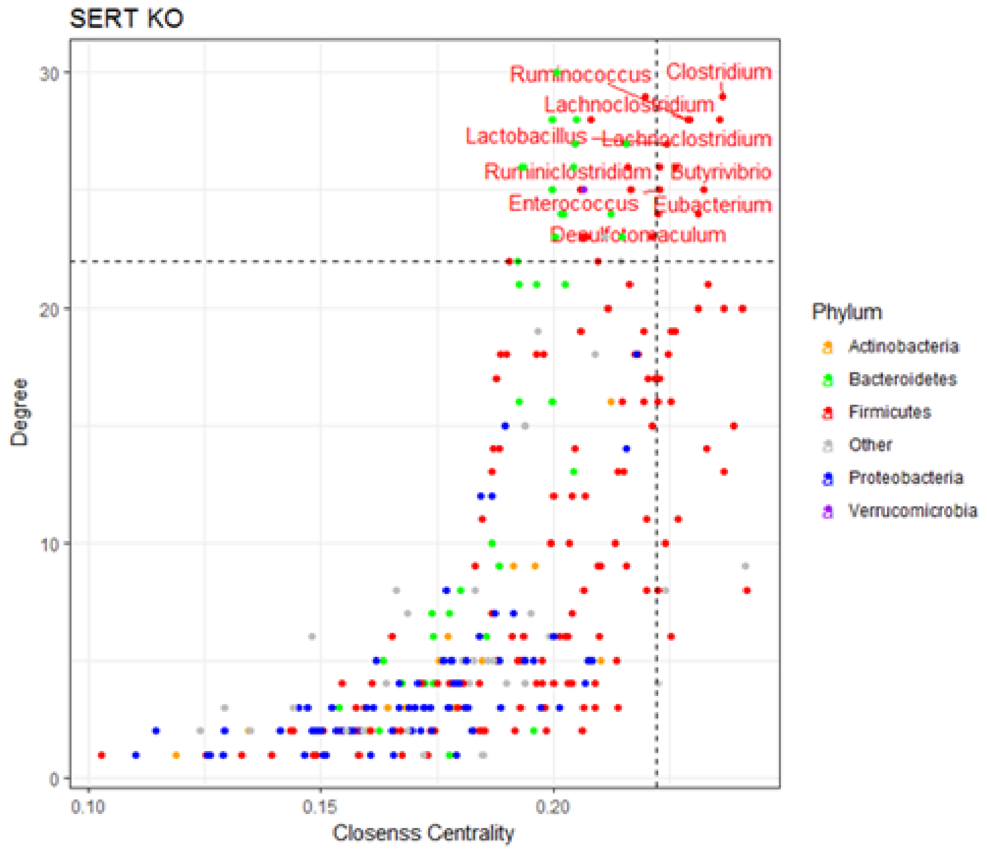
SERT KO
Ruminococcus
Clostridium
Lachnoclostridium
Lactobacillus
Lachnoclostridium
Ruminiclostridium
Butyrivibrio
Enterococcus
Eubacterium
Desulfotomaculum
Degree
Closenss Centrality
Phylum
Actinobacteria
Bacteroidetes
Firmicutes
Other
Proteobacteria
Verrucomicrobia

Figure S2. Differences in hub species between SERT$^{+/+}$ and SERT$^{-/-}$ co-occurrence networks. Scatterplots of species degree and closeness centrality in SERT$^{+/+}$ and SERT$^{-/-}$ co-occurrence networks. Species are colored by phylum. Dashed line represent 90th percentile of Degree and Closeness centrality in each network. Species in the 90th percentile for degree and closeness centrality are labeled by genus.

Table E1: Primer sequences

Gene	Accession No.	Primer sequence (5'→3')
Acot1	NM_012006	F: ATACCCCCTGTGACTATCCTGA<br>R: CAAACACTCACTACCCAACTGT
Hmgcs2	NM_008256	F: GAAGAGAGCGATGCAGGAAAC<br>R: GTCCACATATTGGGCTGGAAA
Hmgcr	NM_008255	F: AGAGCGAGTGCATTAGCAAAG<br>R: GATTGCCATTCCACGAGCTAT
Cyp1a1	NM_001136059	F: GGGTTTGACACAGTCACAACT<br>R: GGGACGAAGGATGAATGCCG
Gapdh	NM_008084	F: TGTGTCCGTCGTGGATCTGA<br>R: CCTGCTTCACCACCTTCTTGAT
Cldn8	NM_018778	F: GCAACCTACGCTCTTCAAATGG<br>R: TTCCCAGCGGTTCTCAAACAC

Table E2. Bacterial Phyla Relative Abundances

	**Cecal**		**Fecal**	
**Phyla**	**SERT$^{+/+}$**	**SERT$^{-/-}$**	**SERT$^{+/+}$**	**SERT$^{-/-}$**
**Firmicutes**	42 (41-57)	56 (52-61)	30 (19-38)	46 (32-61)
**Bacteroidetes**	18 (11-28)	19 (19-20)	46 (32-50)	31 (10-49)
**Proteobacteria**	14 (10-15)	14 (10-16)	11 (8-12)	12 (10-13)
**Actinobacteria**	6 (5-8)	4 (3-5)	8 (4-11)	6 (5-7)
**Verrucomicrobia**	0 (0-5)	0 (0-0)	0 (0-7)	0 (0-0)

Values are expressed as relative abundances in percentages of total reads. Values in parenthesis are interquartile range for the 25th and 75th percentiles. Top five phyla's values are shown.

Tables E3-E6 are provided as individual excel files.
